# A Scale-Adaptive Aggregation and Multi-Domain Feature Fusion Architecture for Small-Target Detection in UAV Aerial Imagery

**DOI:** 10.3390/s26051610

**Published:** 2026-03-04

**Authors:** Zhiwei Sun, Guanglei Zhang, Yuxin Xing, Yuliang Liu

**Affiliations:** 1 College of Artificial Intelligence, Tianjin University of Science and Technology, Tianjin 300457, China; zglei1023@163.com (G.Z.); yx_xing02@163.com (Y.X.); 2 College of Electronic Information and Automation, Tianjin University of Science and Technology, Tianjin 300457, China

**Keywords:** small object detection, aerial monitoring, UAV imagery, scale-adaptive fusion, multi-domain fusion

## Abstract

Vision-based unmanned aerial vehicles (UAVs) have been widely studied and applied in aerial monitoring tasks; however, detecting small objects in UAV imagery remains challenging due to limited visual features, significant scale variations, dense distributions, and complex background interference. In real-world UAV scenarios, small objects often occupy only a few pixels and are easily obscured by cluttered backgrounds, which complicates stable and accurate detection. To address these issues, this study proposes MSCM-YOLO, a UAV-oriented lightweight detection framework based on YOLOv11. The framework integrates four key innovations: (1) a dedicated P2 detection head to preserve high-resolution features for extremely small and dense targets; (2) a lightweight backbone enhanced with Mobile Bottleneck Convolution (MBConv) to improve feature extraction for visually weak objects; (3) a Scale-Adaptive Attention Fusion (SAF) mechanism with a Channel-Adaptive Projection (CAP) module to effectively integrate multi-scale spatial and semantic features under large object-size variations; and (4) a Multi-Domain Feature Attention Fusion (MDFAF) module to enhance target–background discrimination in complex UAV scenes. Experiments on the VisDrone2019 dataset show that MSCM-YOLO achieves mAP50 and mAP50:95 scores of 44.41% and 27.13%, respectively, outperforming the YOLOv11 baseline by 10.77 and 7.22 percentage points. Notably, the proposed framework achieves this significant performance improvement while maintaining a balanced computational profile suitable for UAV deployment. Additional validation on the UAVDT, DIOR, and AI-TOD datasets confirms consistent improvements in mAP50, demonstrating the robustness and generalization ability of the proposed method. Overall, MSCM-YOLO provides an effective and practical solution for accurate small object detection in aerial monitoring applications.

## 1. Introduction

The evolution of unmanned aerial vehicle (UAV) technology, coupled with sophisticated onboard vision sensors, has propelled the widespread adoption of UAV systems in diverse aerial monitoring domains. These domains include urban management, traffic flow analysis, infrastructure inspection, and emergency response operations [1,2,3,4,5,6,7]. UAVs provide operational flexibility, extensive area coverage, and real-time image acquisition. These advantages make UAVs an efficient and economical platform for large-scale aerial surveillance and intelligent environmental perception.

Concurrently, deep-learning-based object detection has advanced rapidly in general scene understanding. CNN-based detectors have set impressive benchmarks on large-scale datasets [8]. Within this landscape, the YOLO family of single-stage detectors has attracted considerable attention in UAV applications, because it balances accuracy and computational cost [9]. However, when applied to UAV imagery, detection performance often drops, especially for small objects. This is largely due to UAV imaging conditions: high flight altitudes and wide fields of view make targets appear extremely small, sometimes spanning only a few pixels. The difficulty is further exacerbated by extreme scale variation, dense target clustering, and complex background interference. As a result, reliable small-object detection remains a major challenge in practical aerial monitoring.

From a computational standpoint, these observed difficulties can be traced to specific shortcomings in conventional detection architectures. First, the limited pixel footprint of small objects makes them highly vulnerable to information loss. Their weak signatures are easily attenuated by repeated convolutional downsampling, which reduces feature activations in deeper layers [10]. Second, the long-standing challenge of effectively fusing high-resolution, detail-rich features from early network stages with the semantically potent but spatially coarse features from later stages persists [11]. The semantic gap between these feature levels often leads to suboptimal fusion. This directly degrades performance on small objects and densely packed targets. Thirdly, prevalent feature refinement and attention approaches often concentrate their efforts on either the spatial or the channel dimension [12], failing to concurrently harness the complementary strengths of frequency-domain analysis [13] and structured positional encoding [14]. This limitation prevents the model from jointly exploiting multi-domain cues. Consequently, subtle textures and structural details are not sufficiently amplified, even though these cues are critical for small-object recognition in complex UAV scenes.

To tackle these interconnected problems, this paper introduces MSCM-YOLO, a detection framework built upon YOLOv11 and explicitly tailored for the small object detection dilemma in UAV contexts. The design of MSCM-YOLO focuses on three aspects: preserving high-resolution visual clues, improving multi-scale feature integration, and enhancing foreground–background separation. Through the incorporation of lightweight network components alongside targeted feature enhancement strategies, the framework seeks to deliver robust detection of small objects while adhering to the computational constraints typical of UAV deployment environments.

The principal innovations presented in this work are outlined below:

(1) Implementation of a Dedicated P2 Detection Branch. We design a new P2 detection head and reconstruct its feature fusion pathway to form a complete P2 branch. This branch enables earlier access to fine-grained details and provides a dedicated stream for very small targets.
(2) Backbone Enhancement via MBConv Integration. The original YOLOv11 backbone is re-engineered by embedding Mobile Inverted Bottleneck Convolution (MBConv) blocks. This redesign reduces computational cost while strengthening the retention of delicate small-object features, thereby alleviating feature degradation.
(3) Development of a Scale-Adaptive Attention Fusion (SAF) Mechanism. We devise the SAF mechanism to orchestrate a more effective amalgamation of features from different network depths [15]. SAF applies dynamic weighting to adaptively narrow semantic gaps between feature levels. Combined with a Channel-Adaptive Projection (CAP) module, it promotes coherent feature flow and improves detection across object scales.
(4) Conception of a Multi-Domain Feature Attention Fusion (MDFAF) Module. This module is engineered to jointly harness and refine discriminative information from three distinct yet complementary realms: spatial granularity, frequency-domain context, and explicit positional relations. By integrating frequency cues with positional information, MDFAF enhances local textures and structural details while maintaining global context [16]. This yields a more robust representation for small targets in cluttered UAV scenes.

## 2. Related Work

### 2.1. Object Detection Algorithms

Object detection is a fundamental research topic in computer vision and has experienced rapid development with the progress of deep learning. According to different architectural paradigms, existing object detection approaches are commonly divided into two-stage and single-stage detectors, which differ in both representation strategy and computational characteristics [17,18,19,20].

Two-stage detectors, exemplified by the R-CNN family, follow a sequential processing pipeline [19]. These methods first generate candidate object regions and then perform region-wise classification and bounding box refinement. By explicitly separating foreground objects from background regions, two-stage detectors typically achieve strong localization accuracy. However, the reliance on multiple processing stages introduces substantial computational overhead and limits inference efficiency, making such approaches less suitable for real-time UAV aerial applications where computational resources and latency are constrained.

Single-stage detectors, in contrast, directly predict object categories and bounding boxes from dense feature maps in a unified forward pass. Representative methods include SSD [18], RetinaNet [20], and the YOLO series [21]. Among them, YOLO-based detectors are particularly attractive for practical deployment due to their favorable balance between detection accuracy and inference speed. By formulating detection as a grid-based, center-aware prediction task, YOLO eliminates the need for explicit region proposal generation and enables efficient end-to-end training [22]. These characteristics have led to the widespread adoption of YOLO as a baseline framework for UAV and aerial image analysis.

### 2.2. Deep Learning Methods for UAV Aerial Small Object Detection

Small object detection in UAV imagery has become a key research focus due to its role in aerial monitoring and intelligent perception. Unlike natural scenes, UAV targets are often only a few pixels and densely packed in cluttered backgrounds. To facilitate research in this area, several dedicated benchmark datasets have been introduced. Du et al. released the VisDrone dataset, which offers large-scale annotated UAV imagery and systematically illustrates the practical difficulties of small object detection in real aerial environments [23]. Similarly, Ge et al. proposed the AI-TOD benchmark, focusing specifically on tiny objects in aerial views and revealing the performance bottlenecks of standard detectors when handling extreme small targets [24]. Xu et al. further dissected the challenges of tiny object detection, noting that extremely limited pixel evidence makes it difficult to learn discriminative features, and that tiny objects are highly sensitive to localization deviations, leading to insufficient supervision during training [25]. Collectively, these benchmarks provide an essential experimental foundation for advancing UAV small object detection.

In response to these challenges, recent research has largely concentrated on improving multi-scale feature representation. Wang et al. proposed an efficient feature fusion framework for UAV object detection that boosts the recall of small objects through optimized cross-scale feature aggregation [26]. Du et al. introduced a cross-layer feature pyramid transformer, which employs attention mechanisms to enhance inter-layer feature interaction and improve small object detection [27]. Li et al. developed a dynamic scale-sequence fusion approach that incorporates adaptive attention to better handle densely distributed small targets, thereby enhancing detection accuracy in difficult aerial settings [28].

Beyond multi-scale fusion, attention-based mechanisms have been widely explored to amplify small object cues. Teng et al. proposed an attention-guided detection network that selectively accentuates regions containing small objects while suppressing background distractions, increasing robustness in cluttered aerial scenes [29]. Liang et al. designed a spatial-channel attention network tailored for UAV imagery, which strengthens the weak activations of small targets through coupled spatial and channel refinement [30]. More recently, TAF-YOLO incorporated early fusion strategies designed for UAV imagery, improving detection robustness and precision via customized feature integration [31].

In parallel, recent YOLO-style detectors have started to explicitly exploit frequency-domain cues during feature fusion to enhance tiny-object perception under cluttered backgrounds. For example, MS-YOLOv11 integrates wavelet-based transformations to decompose features into sub-bands, thereby preserving high-frequency structures (e.g., edges and textures) while maintaining multi-scale semantic context, and further strengthens cross-scale aggregation to improve small object detection performance [32]. Similarly, an improved YOLOv11 framework introduces a frequency–spatial feature extraction and fusion module that adopts a parallel multi-branch design to hierarchically fuse frequency-domain components with spatial features, enhancing robustness in dense small-object scenes with complex backgrounds [33]. Beyond wavelet-style fusion, a YOLOv12-based detector incorporates a frequency-domain self-attention solver, which shifts attention computation to the Fourier domain via FFT-based operations and maps the result back through inverse FFT, enabling efficient global dependency modeling while leveraging low-/high-frequency information [34].

Notwithstanding these advances, a common limitation persists: many existing methods lack a unified framework that jointly models spatial detail, semantic context, frequency-domain information, and explicit positional relations. This gap restricts the capacity to form robust representations of small objects within the complex visual environments characteristic of UAV imagery [35].

## 3. Methods for Implementation

### 3.1. Overview of MSCM-YOLO Model

**Figure 1:**
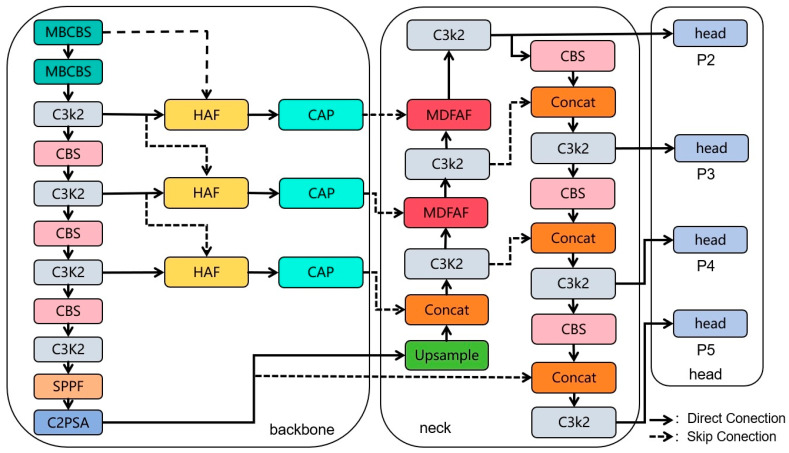
Structure of the MSCM-YOLO network.

Small object detection in unmanned aerial vehicle (UAV) imagery remains particularly challenging. Targets typically occupy only a few pixels, present weak semantic cues, and are prone to occlusion in complex aerial environments. Moreover, background clutter and large scale variations further blur the distinction between targets and surroundings. This increases localization ambiguity and raises the risk of missed detections. Under such conditions, conventional convolutional architectures tend to suppress subtle yet discriminative features during background suppression and multi-scale downsampling, thereby reducing the representational fidelity of small objects. To address these limitations, we propose a UAV-oriented detection framework, MSCM-YOLO, which alleviates small-object feature attenuation by strengthening multi-scale feature interaction and context-aware fusion, thus preserving fine-grained cues under cluttered backgrounds and occlusion. The overall architecture is illustrated in Figure 1.

To address insufficient high-resolution feature utilization and weak small-target representation in existing UAV detection frameworks—particularly critical when targets occupy only a few pixels—we introduce a dedicated P2 detection head to preserve fine-grained spatial details and enable high-resolution features to directly participate in prediction, enhancing sensitivity to extremely small targets. To improve the extraction of weak visual patterns prone to attenuation during deep convolutional processing, Mobile Bottleneck Convolution (MBConv) is incorporated into the backbone to strengthen local feature representation while maintaining computational efficiency. Moreover, to alleviate inefficient cross-level feature integration caused by semantic discrepancies between shallow and deep layers, a Scale-Adaptive Attention Fusion (SAF) mechanism combined with a Channel-Adaptive Projection (CAP) module is designed to adaptively align and fuse low-level spatial details with high-level semantic information. Finally, to enhance target–background discrimination in complex aerial environments, a Multi-Domain Feature Attention Fusion (MDFAF) module is introduced to jointly exploit spatial, frequency, and positional cues, reinforcing small-object representations and improving detection robustness.

### 3.2. Improvements in Backbone Structure

In UAV small object detection tasks, the detection accuracy of small targets is typically much lower than that of large-scale objects, mainly due to limitations in feature extraction and fusion mechanisms. In backbone networks, sequential convolution and downsampling operations fail to preserve the limited pixel information of small targets, leading to severe loss of fine-grained spatial details. Meanwhile, conventional feature pyramid fusion strategies are often overly simplistic and lack selective enhancement of critical features, causing low-level information to be overwhelmed by background interference. In addition, semantic discrepancies across feature layers hinder effective integration of spatial details and high-level semantics, further restricting the representational capacity of small objects. These factors collectively contribute to the suboptimal performance of existing methods in UAV small object detection.

**Figure 2:**
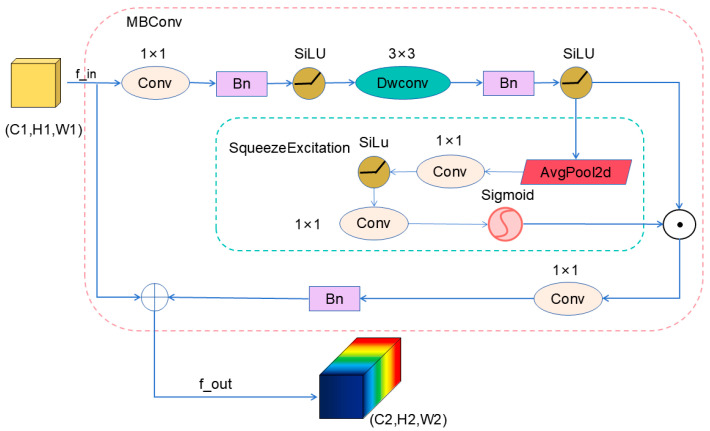
Schematic diagram of MBConv module.

(1) To alleviate the bottleneck of insufficient feature extraction capabilities, this paper first employs the MBConv module as the foundational building block. Its unique inverted residual structure and attention mechanism significantly enhance feature representation capabilities while ensuring computational efficiency. The expand–compress channel transformation strategy effectively boosts feature response for small objects. The architecture of the MBConv module is illustrated in Figure 2.

This module significantly reduces the parameter and computational overhead of initial feature extraction through deep separable convolutions, while its “expand–compress” inverse residual structure ensures rich feature representations. This paper configures moderate expansion ratios for these shallow MBConv layers, prioritizing the retention of spatial details critical for small object detection while controlling computational complexity. This design enables the network to efficiently capture discriminative features of minute objects—such as edges and corners—at the input stage, establishing a lightweight and effective foundation for subsequent feature fusion. This achieves an optimal balance between computational efficiency and detail preservation.

Given an input feature map X ∈ R^H×W×Cin^, the MBConv module first increases the channel dimension through an expansion stage:

(1) $$F_{\exp}=\mathrm{SiLU}\left(\mathrm{BN}\left({\mathrm{Conv}}_{1\times 1}(F_{\mathrm{in}})\right)\right)$$

where C_exp_ = C_in_ × t represents the expanded number of channels, with t denoting the expansion ratio. In the experiments conducted for this study, t was set to 3, resulting in the expanded number of channels being calculated as C_in_ × 3.

The expanded features are subsequently fed into the deep convolutional stage:

(2) $$\begin{array}{c} X_{dw}=F_{dw}(X_{exp})=SiLU(BN({Conv}_{k\times k}^{depthwise}(X_{exp}))) \end{array}$$

Conv_K×K_^Depthwise^ denotes a depthwise convolution with a kernel size of k × k, performing spatial convolutions independently on each input channel. This approach significantly reduces computational complexity and the number of parameters.

The features from the deep convolutional layer are then fed into the feature compression module. This module first performs spatial compression via a global average pooling operation, generating a channel descriptor Z ∈ R^1×1×Cexp^:

(3) $$\begin{array}{c} Z=GAP(X_{dw}) \end{array}$$

Then, channel attention weights are generated through two fully connected layers (implemented as 1 × 1 convolutions in the code) and activation functions:

(4) $$\begin{array}{c} W=\sigma ({Conv}_{1\times 1}(SiLU({Conv}_{1\times 1}(Z)))) \end{array}$$

The first Conv_1×1_ layer reduces the number of channels by a factor of r (where r is the compression ratio). The second Conv_1×1_ layer restores the original number of channels, with σ being the Sigmoid function, generating channel weights within the range [0,1].

(5) $$\begin{array}{c} X_{se}=F_{se}(X_{dw})=W⊙X_{dw} \end{array}$$

Here, ⊙ denotes channel-wise multiplication between the attention weight tensor W and the feature map Xdw.

Finally, the features are projected back to the target dimension through the compression stage:

(6) $$\begin{array}{c} X_{proj}=F_{proj}(X_{se})=BN({Conv}_{1\times 1}(X_{se})) \end{array}$$

This operation reduces the number of channels from C_exp_ to the target output channel count C_out_ while preserving channel dimensionality, thereby enhancing the network’s expressive power.

When the input and output channel counts are identical and the stride is set to 1, the module employs residual connections and incorporates stochastic depth regularization:

(7) $$\begin{array}{c} X_{out}=X_{proj}+DropConnect(X) \end{array}$$

During training, DropConnect randomly drops entire feature maps with probability *p*.

**Figure 3:**
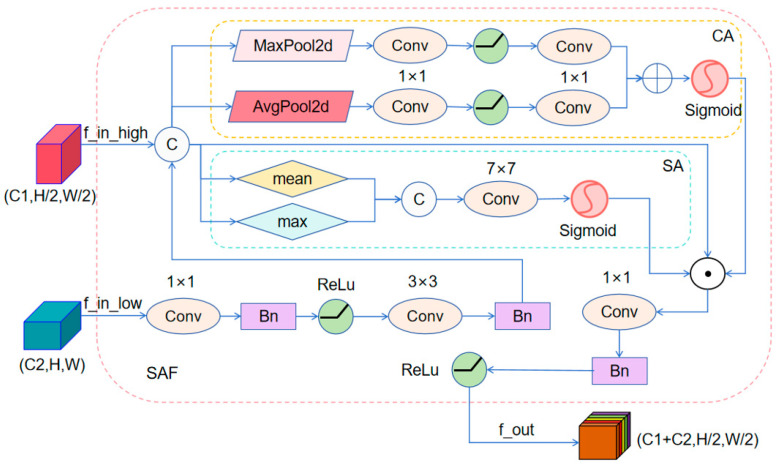
Schematic diagram of SAF module.

(2) To mitigate the limitation of insufficient utilization of low-level detail features in existing models, this paper introduces a Scale-Adaptive Attention Fusion Module (SAF). The module adaptively fuses high-resolution spatial details, mid-scale structural cues, and deep semantic context via a detail-enhancement pathway from shallow to deep layers. The SAF structure is illustrated in Figure 3.

This approach continuously strengthens the extraction and propagation of discriminative features for small targets across different receptive fields, establishing a consistent detail-enhancement pathway throughout the network. This pathway improves the model’s sensitivity to subtle patterns while maintaining stable feature propagation across depth.

The SAF module first maps low-level features to a dimensional space matching high-level features via a channel adjustment layer, then employs a dual-path attention mechanism to enhance features. Channel attention precisely calibrates the weight distribution across feature channels through global context modeling, while spatial attention focuses on discriminative regions within feature maps. The synergistic interaction of these two attention mechanisms enables the model to adaptively preserve the fine-grained spatial information of low-level features while enhancing the semantic expressiveness of high-level features. This scale-adaptive fusion mechanism significantly enhances the model’s feature representation ability for multi-scale objects, demonstrating exceptional adaptability when encountering detection targets with drastic scale variations. It provides feature representations with stronger discriminative power and more balanced spatial-semantic properties for subsequent multi-branch fusion.

Given the concatenated feature map F_cat_ ∈ R^B×(Chigh×2)×Hh×Wh^, where B denotes the batch size, C_high_ represents the number of high-level feature channels, and H_h_ and W_h_ denote the spatial dimensions of the high-level features. First, the channel attention module compresses the spatial dimensions via Global Average Pooling (GAP) to generate channel descriptors. It then learns inter-channel dependencies using a Multi-Layer Perceptron (MLP, composed of two 1 × 1 convolutions), ultimately producing channel attention weights A_c_ through the Sigmoid activation function σ:

(8) $$A_{c}=\sigma (MLP(GAP(F_{cat}))+MLP(MaxPool(F_{cat})))$$

MLP denotes a multi-layer perceptron, while GAP refers to the global average pooling operation.

At the same time, the spatial attention module calculates the mean and maximum values across the channel dimension of the feature map. These values are concatenated and then processed through a 7 × 7 convolution and an activation function to generate the spatial attention weights A_s_:

(9) $$\begin{array}{c} A_{s}=\sigma ({Conv}_{7\times 7}([Mean(F_{cat}),Max(F_{cat})])) \end{array}$$

Here, Mean denotes the calculation of the mean across the channel dimension, Max denotes the calculation of the maximum across the channel dimension, and [·,·] denotes the concatenation operation across the channel dimension. Subsequently, element-wise multiplication ⊙ is used to interact the channel attention and spatial attention weights with the original features, achieving attention enhancement:

(10) $$\begin{array}{c} F_{\mathrm{enh}}=A_{c}⊙F_{\mathrm{cat}}⊙A_{s} \end{array}$$

This operation adaptively highlights important feature regions while suppressing redundant information.

Finally, the enhanced features are fused through a 1 × 1 convolution, batch normalization (BN), and ReLU activation function, yielding the fused feature output F_out_SAF_:

(11) $$\begin{array}{c} F_{out\_SAF}=ReLU(BN({Conv}_{1\times 1}(F_{enh})))\in R^{B\times (C_{low}+C_{high})\times H_{h}\times W_{h}} \end{array}$$

Here, C_low_ denotes the number of low-level feature channels. In this model, the compression ratio r of channel attention is set to 16, ensuring effective integration of multi-scale features and enhanced expressive capability.

**Figure 4:**
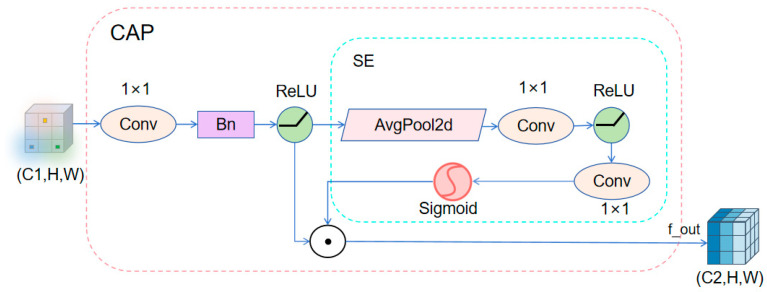
Schematic diagram of CAP module.

(3) To alleviate fusion bottlenecks caused by mismatched feature distributions and channel dimensions across different branches, this study proposes a Channel-Adaptive Projection (CAP) module, as illustrated in Figure 4. Centered on a learnable 1 × 1 convolution, the CAP module unifies channel dimensions of cross-source features, reducing semantic inconsistency and response misalignment during fusion. Unlike conventional projection operations, CAP integrates channel transformation with a compression–excitation mechanism, introducing global context-aware channel attention to selectively enhance informative features while suppressing redundant responses.

This module is designed to strike a balance between lightweight implementation and practical utility. The projection component introduces only a minimal set of parameters to control computational overhead, while the embedded attention mechanism aims to enhance the model’s ability to perceive discriminative features. Preliminary validation on small object detection tasks for drones suggests that this module may help improve the retention of fine-grained structural and edge information, thereby providing subsequent detection modules with higher-quality, multi-scale feature inputs.

Upon receiving the output from the upper-level SAF module, the CAP module first performs channel projection on the input features through 1 × 1 convolution, batch normalization, and ReLU activation, followed by global average pooling to compress spatial information. Two fully connected layers (implemented as 1 × 1 convolutions) with Sigmoid activation then generate channel attention weights. Finally, multiplying these attention weights with the projected features achieves adaptive feature enhancement, yielding F_out_CAP_:

(12) $$\begin{array}{cc} F_{out\_CAP}= & ReLU(BN({Conv}_{1\times 1}(F_{in})))⊙\\ & \sigma ({Conv}_{1\times 1}(ReLU({Conv}_{1\times 1}(GAP(ReLU(BN({Conv}_{1\times 1}(F_{in})))))))) \end{array}$$

where F_in_ ∈ R^B×Cin×H×W^ is the output of the preceding layer SAF.

### 3.3. Improvements in Neck Structure

**Figure 5:**
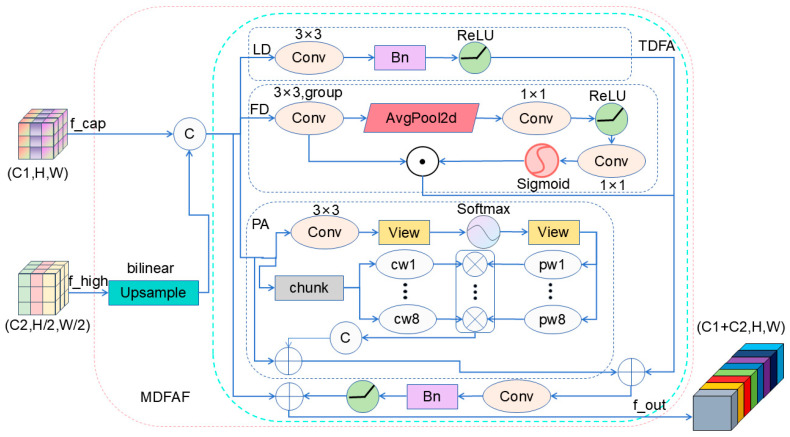
Schematic diagram of MDFAF module.

This paper proposes a Multi-Domain Feature Attention Fusion (MDFAF) module designed to complement existing spatial–frequency domain fusion methods and address the lack of explicit positional awareness modeling, which makes it difficult to accurately pinpoint the spatial locations of small targets amid complex background clutter. By coordinating spatial, frequency, and positional attention branches, the proposed module enhances the integration of global structural information and positional cues while preserving local detail features. Specifically, the spatial branch captures local texture characteristics, the frequency-aware branch captures global contextual cues via grouped filtering and channel-wise modulation, providing complementary global information to the spatial-detail branch, and the positional branch improves spatial sensitivity via grouped convolutions. The collaborative operation of these branches effectively mitigates semantic dilution within the feature pyramid. By leveraging complementary multi-domain features, MDFAF enhances contextual representation and localization accuracy for small objects. The overall structure of MDFAF is illustrated in Figure 5.

This module addresses the limitation of single-domain feature representation in complex scenarios through multi-domain collaboration. For UAV small-object detection, the module enhances recognition of minute targets via frequency-aware feature modulation, while improving localization accuracy through explicit position-aware attention.

Given input feature maps F_f_cap_ ∈ R^B×C1×H1×W1^ and F_f_high_ ∈ R^B×C2×H2×W2^, the module first adjusts the feature maps to a uniform spatial dimension through an upsampling operation:

(13) $$\begin{array}{c} H_{target}=\max(H_{1},H_{2}),W_{target}=\max(W_{1},W_{2}) \end{array}$$

(14) $$\begin{array}{c} Upsample(F)=BilinearInterpolate(F,(H_{target},W_{target})) \end{array}$$

BilinearInterpolate denotes the bilinear interpolation upsampling operation. The upsampled features are concatenated and merged into F_cat_ ∈ R^B×(C1+C2)×Htarget×Wtarget^.

The signal then enters the Triple Domain Feature Attention (TDFA) module, which comprises three parallel branches: the spatial domain branch (LD), the frequency domain branch (FD), and the position-aware branch (PA).

The LD branch extracts local detail features using a 3 × 3 convolution:

(15) $$\begin{array}{c} F_{out\_LD}=ReLU(BN({Conv}_{3\times 3}(F_{cat}))) \end{array}$$

The FD branch analyzes frequency-domain features through grouped convolutions and a compressed activation mechanism:

(16) $$\begin{array}{c} F_{out\_FD}=F_{cat}⊙\sigma ({Conv}_{1\times 1}(ReLU({Conv}_{1\times 1}(GAP({Conv}_{3\times 3}^{group}(F_{cat})))))) \end{array}$$

Conv_3×3_^group^ denotes grouped convolutions.

The PA branch captures spatial position information through a grouped attention mechanism:

(17) $$\begin{array}{c} F_{out\_PA}=F_{in}+\gamma \cdot Concat{\left[F_{in}^{\left(i\right)}⊙Softmax({Conv}_{1\times 1}(F_{in}){)}^{\left(i\right)}\right]}_{i=1}^{G} \end{array}$$

The PA branch captures spatial positional information through a grouped attention mechanism. Specifically, the input feature map is divided into G groups. For each group, spatial attention weights are computed via a 1 × 1 convolution followed by Softmax normalization. These weights are then multiplied with the corresponding group features and concatenated, with a learnable scaling parameter γ controlling the fusion strength.

Then, the triple-domain features are integrated through fusion convolutions, and the enhanced features are output via residual connections:

(18) $$\begin{array}{c} F_{out\_TDFA}=F_{in}+\gamma \cdot Fusion(F_{out\_LD},F_{out\_FD},F_{out\_PA}) \end{array}$$

Here, Fusion denotes the feature fusion operation achieved through 1 × 1 convolutions. The Fusion operation concatenates the three feature maps F_out_LD_, F_out_FD_, F_out_PA_ along the channel dimension, then applies a 1 × 1 convolution to integrate them.

In Equations (17) and (18), γ denotes the same learnable scaling parameter that modulates the contribution of the attention-enhanced features.

The final output of MDFAF is:

(19) $$\begin{array}{c} F_{out\_MDFAF}=TDFA(Concat(Upsample(F_{f\_cap}),Upsample(F_{f\_high}))) \end{array}$$

The MDFAF module achieves adaptive fusion of multi-scale features through a triple attention mechanism across spatial, frequency, and position domains, significantly enhancing the model’s ability to model complex scenes.

### 3.4. Improvements in Head Structure

In the detection head section, the original three-detection-head structure of the base model exhibits inherent limitations when processing small targets with extreme scale variations in remote sensing imagery. For numerous extremely small targets, the receptive field of the highest-resolution P3 layer remains excessively large, causing subtle yet critical texture features to be diluted during propagation and resulting in missed detections. This model expands the detection heads to four, introducing a higher-resolution P2-layer detection head to construct a denser scale pyramid from P2 to P5 [36]. This enhancement enables the network to capture pixel-level details directly at earlier feature layers, providing a dedicated detection dimension for minute targets and improving inspection capabilities for densely packed small objects. Simultaneously, this paper reconstructs the corresponding feature fusion pathway, forming a shorter, more efficient low-layer feature transmission channel that ensures high-resolution details are fully preserved and utilized for precise localization [37]. These enhancements significantly boost the model’s ability to capture extremely scale-imbalanced targets in drone scenarios, demonstrating particularly notable improvements in recall and localization accuracy for densely packed, minute objects.

## 4. Experiment

### 4.1. Datasets

**Figure 6:**
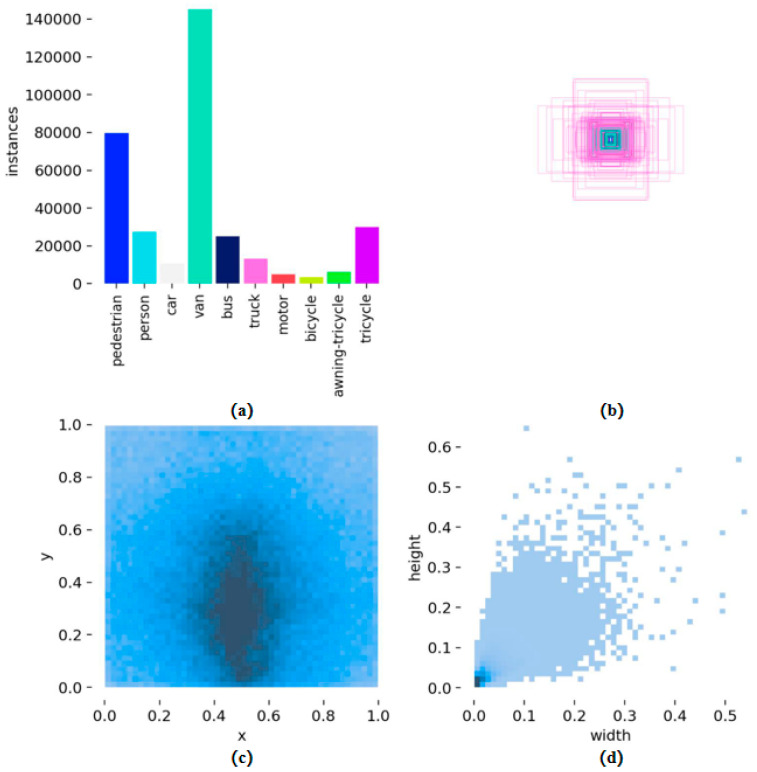
Visualization of training dataset. (**a**) Category distribution. (**b**) Distribution of label frame length and width. (**c**) Spatial distribution of object centers. (**d**) Distribution of object width and height.

The VisDrone2019 dataset [23] is a large-scale benchmark dataset widely used in drone vision research. With image resolutions up to 3054 × 1586, it comprises 6471 training images and 548 validation images, totaling 343,205 annotated instances. Due to the aerial perspective captured by drones, each image contains an average of 53 object instances, with the vast majority smaller than 32 × 32 pixels—representing a typical dense small object detection scenario. The dataset defines 10 object categories including pedestrians, vehicles, and bicycles, spanning diverse complex environments such as urban areas, rural landscapes, and highways. These characteristics of high density, multi-scale, and small objects make it an ideal benchmark for evaluating the robustness of small object detection algorithms. Figure 6 illustrates the dataset’s category distribution, bounding box size distribution, spatial distribution characteristics, and object size variation. It clearly reveals severe category imbalance, where car instances vastly outnumber other categories. Simultaneously, the overwhelming majority of objects fall within the micro-object size range, posing significant challenges to a detector’s feature extraction and localization capabilities.

### 4.2. Implementation Details

All experiments in this study were conducted on the same server to ensure consistency in the experimental environment. The specific hardware and software configurations are as follows: the operating system is Ubuntu 20.04, the central processing unit (CPU) is an Intel^®^ Xeon^®^ Silver 4210R, and the graphics processing unit (GPU) is a single NVIDIA GeForce RTX 3090 (24GB VRAM). For the software environment, a training platform was built based on CUDA 12.2 and the PyTorch (Torch 2.5.1) deep learning framework, with Python 3.12 serving as the programming language, see Table 1.

All ablation variants and the proposed MSCM-YOLO are trained and evaluated using the same dataset split, input resolution, optimizer, and training schedule (Table 2). We adopt Ultralytics YOLOv11n as the baseline (Variant A in Table 3; 2.59M parameters in our implementation) and follow the controlled-variable principle. To avoid confounding factors, we use a unified training pipeline and apply the same data augmentation settings to all variants. Moreover, all models are trained from randomly initialized weights (i.e., without loading external pre-trained weights) to eliminate the influence of pretraining and enable a cleaner assessment of the proposed modules.

### 4.3. Evaluation Metrics

To objectively evaluate the detection performance of the proposed model, we adopt a set of standard metrics commonly used in object detection benchmarks. Following the evaluation protocol of the MS COCO benchmark proposed by Lin et al. [38], we report precision (P), recall (R), F1-score, Average Precision (AP), mean Average Precision (mAP), as well as mAP at IoU = 0.50 (mAP50) and the averaged mAP over IoU thresholds from 0.50 to 0.95 with a step size of 0.05 (mAP50:95).

### 4.4. Ablation Study

To evaluate each component’s contribution, we conducted an ablation study on VisDrone2019 using YOLOv11 as the baseline (Variant A). Four enhancements were assessed: the P2 head, MBConv, the combined SAF+CAP module (where CAP projects SAF-fused features for better integration; SAF and CAP are treated as a single component), and MDFAF. All models were trained from scratch under identical conditions for a fair comparison.

The contribution of each module is quantified through comprehensive evaluation metrics, including Precision (P), Recall (R), mAP50, mAP50:95, number of parameters (Param), and inference speed (FPS). Detailed ablation results are presented in Table 3, where “√” indicates the introduction of the corresponding module.

#### 4.4.1. Impact of Individual Components

The individual contribution of each module is clearly demonstrated when sequentially integrating them into the P2-augmented baseline (Variant B). Beginning with Variant B, the dedicated P2 detection head alone elevates mAP@50 by 3.83 percentage points over the original baseline (Variant A), unequivocally underscoring the indispensable role of preserving high-resolution, fine-grained details for small-object detection. Subsequently, incorporating the MBConv block (Variant C) yields a modest yet parameter-free recall improvement (+0.26 pp), affirming its effectiveness in lightweight and efficient feature representation. In stark contrast, the SAF+CAP module (Variant D) delivers the most substantial gains in both recall (+2.41 pp) and mAP@50 (+1.97 pp), solidly validating its sophisticated design for adaptive, scale-adaptive fusion across semantic levels. Most notably, the MDFAF module (Variant E) provides the most comprehensive performance leap, boosting mAP@50 and precision by 5.36 pp and 4.36 pp, respectively. This emphatically highlights the superior value of synergistically integrating complementary cues from the spatial, frequency, and positional domains, which collectively enhance the model’s discriminative power in complex aerial scenes.

#### 4.4.2. Synergistic Effects of Module Combinations

Analyzing the combinations of modules reveals significant synergistic interactions. Specifically, the model integrating both SAF+CAP and MDFAF (Variant H) achieves an mAP@50 of 44.18%, outperforming the configurations with either module alone (Variants D and E). This demonstrates the complementary roles of cross-level spatial–semantic fusion (SAF+CAP) and multi-domain feature enhancement (MDFAF). The full model, Variant I (MSCM-YOLO), which incorporates all four proposed components, attains the best overall performance. With a mAP@50 of 44.41%, it delivers a substantial improvement of 10.77 percentage points over the baseline (Variant A), conclusively validating the cumulative and synergistic efficacy of the complete architecture.

#### 4.4.3. Analysis of Efficiency Trade-Off

The ablation study further elucidates the inherent performance-efficiency trade-off in the proposed architecture. As quantitatively detailed in Table 3, the MBConv module provides a lightweight, parameter-efficient enhancement, delivering an initial performance improvement with only a minimal increase in model complexity. In contrast, the more sophisticated SAF+CAP and MDFAF modules introduce additional computational overhead through their multi-level and multi-domain operations. Consequently, the final model (Variant I) maintains a compact parameter footprint of 3.97M, but the cumulative cost of the added modules leads to a pronounced reduction in inference throughput.

Specifically, the inference speed decreases from 598.73 FPS in the baseline (Variant A) to 167.81 FPS in the final configuration (Variant I). Due to its multi-branch architecture and frequency-domain transformations, MDFAF incurs the highest computational cost. In future work, MDFAF can be lightweighted through pruning and distillation to build a lightweight substitute module for MDFAF. All speed measurements were obtained under strictly controlled, identical conditions (RTX 3090 GPU, 640 × 640 input) to ensure direct comparability and reproducibility.

Although this speed reduction is substantial, the final throughput remains within a practical range for many real-time UAV detection applications [39], particularly those where accuracy under complex scenes is paramount. In such scenarios—characterized by dense small objects, occlusions, and scale variation—detection accuracy often constitutes the primary deployment bottleneck. From this perspective, the observed accuracy gains provide strong justification for the accepted computational cost in accuracy-critical settings.

To evaluate its practical deployment potential on resource-constrained UAV onboard platforms, we combine the benchmark performance with the theoretical computational capability of embedded devices to extrapolate the expected runtime. The analysis indicates that on a typical edge computing platform, Jetson Xavier NX, the model is expected to achieve an inference speed of over 40 FPS, which is generally sufficient for real-time monitoring tasks. On the more compute-limited Jetson Nano, the expected speed is around 20 FPS, still viable for certain near-real-time applications or with further optimization.

Overall, MSCM-YOLO embodies a deliberate design prioritization: it strategically trades part of the baseline’s extreme inference speed for substantially improved detection accuracy and robustness, while preserving a moderate model size. A comprehensive comparison of its accuracy-efficiency Pareto frontier against contemporary detectors is provided in Section 4.2.

### 4.5. Comparisons with Other Object Detection Algorithms

To further validate the superiority of the proposed MSCM-YOLO, this section presents a comprehensive comparison with a wide spectrum of state-of-the-art object detection models on the VisDrone2019 benchmark. These include anchor-free detectors such as ATSS [40], TOOD [41], and VFNet [42]; various YOLO-based architectures and their enhanced variants, including Gold-YOLO [43], HRMamba-YOLO [44], DASSF-YOLO [28], RMH-YOLOv8n [45], SRM-YOLO [46]; as well as the transformer-based detector RT-DETR-R18 [47]. All methods are compared on the same VisDrone2019 benchmark and evaluated using the same metrics. For YOLO-family baselines, we re-train them under the unified setting in Table 2; for other detectors, we report results from their official implementations or the original papers when a unified re-training pipeline is not available.

The VisDrone2019 dataset comprises 10 categories: Pedestrian (Ped), People (Peo), Bicycle (Bic), Car, Van, Truck, Tricycle (Tri),Awning-Tricycle (Awn), Bus, and Motor. Its complex scenes and dense small objects pose significant challenges to detection capabilities. The following table details the average precision for each model across all categories and the overall mean average precision. Detailed experimental results are presented in Table 4.

#### 4.5.1. Comparison with Lightweight YOLO Models

Compared to the lightweight YOLO baselines with similar design philosophies, MSCM-YOLO demonstrates a substantial advantage. It surpasses YOLOv8, YOLOv11, and YOLOv12 by 10.6, 10.8, and 12.5 percentage points in overall mAP50, respectively. Notably, it also outperforms other efficient variants such as RMH-YOLOv8n (42.4% mAP50 with 1.3M parameters) and SRM-YOLO (39.4% mAP50 with 3.2M parameters). Although our model has slightly more parameters (3.9M), the significant accuracy gain validates the effectiveness of our architectural innovations in exchanging a modest complexity increase for a major performance boost.

#### 4.5.2. Comparison with High-Performance Detectors

When compared against larger, high-performance detectors, MSCM-YOLO remains highly competitive. It outperforms complex anchor-free models like TOOD (41.0%) and VFNet (41.3%), while using only about one-tenth of their parameters. Furthermore, our model exceeds the performance of the recent HRMamba-YOLO (38.9%) and DASSF-YOLO (39.6%), which are also tailored for UAV imagery. Most notably, MSCM-YOLO achieves a higher mAP50 than the efficient transformer-based RT-DETR-R18 (42.5%), which has approximately five times more parameters (19.9M). This clearly highlights the efficiency and precision of our multi-domain feature fusion approach.

#### 4.5.3. Per-Category Performance Analysis

The per-category results confirm the robustness of MSCM-YOLO across object types and scales. It achieves the best or competitive accuracy in 9 out of 10 categories. Particularly for key small-object categories like Ped (50.7%), Peo (41.2%), and Motor (51.7%), our model delivers top-tier performance. It also excels in detecting larger but crucial categories such as Car (83.6%) and Bus (61.8%). For challenging, sparsely represented categories like Bic and Awn, MSCM-YOLO maintains competent detection rates, demonstrating its balanced capability.

#### 4.5.4. Overall Performance and Efficiency Comparison

To provide a concise overview of the overall efficiency–accuracy trade-off, Table 5 contrasts MSCM-YOLO against several recent and competitive models specifically designed or widely used for UAV and small object detection. The selected competitors represent a range of design philosophies, from lightweight adaptations like RMH-YOLOv8n and SRM-YOLO to more complex architectures like HRMamba-YOLO, allowing for a direct evaluation of whether our architectural innovations yield superior overall performance per unit of computational cost.

**Figure 7:**
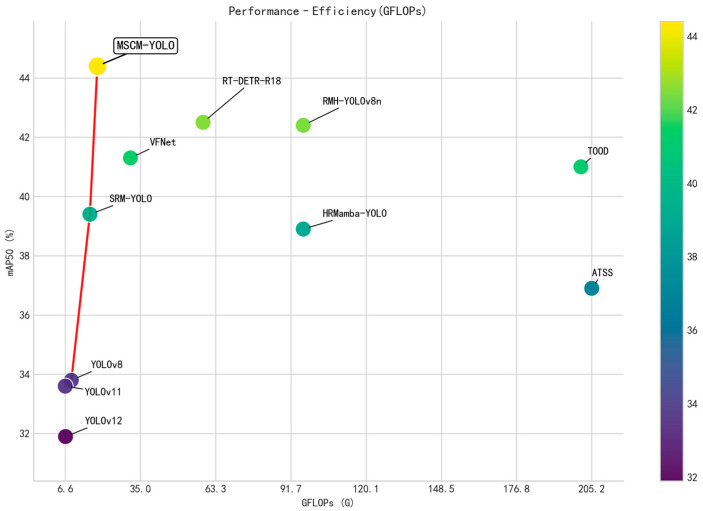
Performance–complexity comparison of different detectors.

Notably, MSCM-YOLO achieves superior detection accuracy with a remarkably compact model architecture, requiring only 3.9M parameters and 18.7 GFLOPs. As presented in Table 3, it attains the best overall accuracy while maintaining low parameter counts and computational overhead, highlighting an excellent performance-efficiency trade-off. The performance-complexity scatter plot (Figure 7) further confirms that MSCM-YOLO is situated on the Pareto frontier, near the ideal upper-left corner. Specifically, compared to SRM-YOLO, MSCM-YOLO delivers a significant accuracy gain with only a moderate increase in GFLOPs, indicating a higher marginal return on additional computation and effectively pushing the Pareto frontier upward. In contrast, several competitors incur substantially higher computational costs without matching MSCM-YOLO’s accuracy, rendering them Pareto-dominated. Collectively, these results demonstrate that MSCM-YOLO achieves enhanced accuracy while preserving high computational efficiency. This balance makes it particularly suitable for deployment on resource-constrained edge devices.

### 4.6. Generalization Experiments

To validate the robustness and generalization capability of MSCM-YOLO, we further conduct experiments on three additional benchmarks beyond VisDrone2019, including UAVDT [48], DIOR [49], and AI-TOD [24]. These datasets cover more extreme UAV traffic scenes (UAVDT) and broader remote-sensing scenarios with diverse object categories (DIOR) as well as tiny-object-focused settings (AI-TOD). For each dataset, we follow its official train/val (or train/test) split and train the models on the training set before evaluation, using the same input size and evaluation metrics as defined in Section 4.2 and Section 4.3.

- UAVDT: Focuses on UAV-view vehicle detection in urban traffic scenes. It features dense vehicles, viewpoint/scale variations, frequent occlusion, motion blur, and complex backgrounds under diverse lighting and weather conditions, making detection a persistent challenge in real-world UAV surveillance.
- DIOR: A large-scale dataset with 20 common object categories in diverse optical remote sensing scenes, evaluating generalization across multiple land-cover types and more complex semantic contexts.
- AI-TOD: Specifically designed for tiny object detection in aerial images, where targets occupy an average of only 0.12% of the image area, testing the limit of feature extraction capability.

#### 4.6.1. Results on UAVDT

The UAVDT dataset focuses on three vehicle categories, namely Car, Truck, and Bus, captured from UAV perspectives under complex traffic scenes. In Figure 7, Car, Truck, and Bus denote the category-wise detection performance (AP@0.5) for each class, while mAP50 (%) represents the overall AP@0.5 averaged across all categories. In addition, model size and computational complexity are reported using parameter count and GFLOPs, respectively, to reflect the efficiency of different detectors.

**Figure 8:**
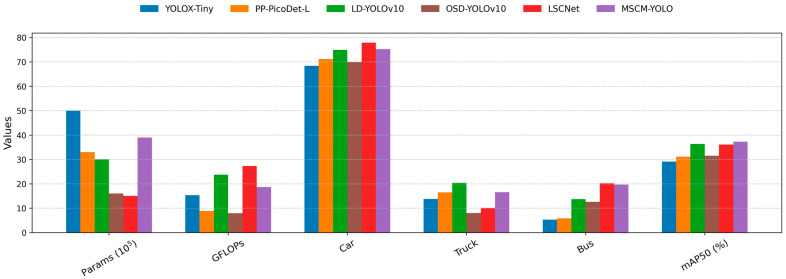
Performance comparison of different models on the UAVDT dataset.

Figure 8 presents a comparative evaluation of MSCM-YOLO against five representative lightweight detectors for UAV applications: YOLOX-Tiny, PP-PicoDet-L [50], LD-YOLOv10 [51], OSD-YOLOv10 [52], and LSCNet [53].

The proposed MSCM-YOLO achieves the highest overall detection accuracy, securing the top mAP50 (%) score among all methods. It demonstrates consistently robust performance across object categories under varied UAV viewpoints and scale changes. While remaining highly competitive for common categories like Car, MSCM-YOLO exhibits a clear advantage in detecting more challenging classes such as Truck and Bus. These categories are particularly difficult in UAV imagery due to smaller target sizes and complex backgrounds, highlighting the strength of our approach in practical scenarios.

Nevertheless, this accuracy gain entails a trade-off in computational efficiency. As indicated by the GFLOPs and parameter count (×10^5^), MSCM-YOLO requires more computational resources and has a larger model size than ultra-lightweight alternatives (e.g., YOLOX-Tiny and PP-PicoDet-L). This implies that deploying our model on resource-constrained UAV edge devices requires careful consideration of hardware capabilities and real-time latency constraints in field operations and dynamic operational environments. Despite its increased complexity, the superior performance of MSCM-YOLO on challenging targets makes it a suitable and compelling choice for missions where detection reliability and accuracy are critical, even if they come at a higher computational cost in practical deployments.

#### 4.6.2. Results on DIOR and AI-TOD

To further assess cross-domain adaptability, we compare MSCM-YOLO with a range of representative models on two widely used remote sensing benchmarks: DIOR, which covers 20 common object categories in diverse scenes, and AI-TOD, which is focused on minute object detection. The compared models include the YOLO-series baselines YOLOv8, YOLOv11, and YOLOv12; other YOLO-based variants such as FFCA-YOLO [54], DASSF-YOLO, and CIMB-YOLO [55]; the anchor-free detector ATSS; as well as recent specialized models including HGNetv2 [47], LeYOLO [56], and HiDRA-DCDNet [57]. The quantitative results are summarized in Table 6.

Based on the results in Table 6, the proposed MSCM-YOLO demonstrates significant advantages across three key metrics: F1 score, mAP50, and mAP50:95. On the DIOR dataset, our model improves mAP50 by 2.9 percentage points compared to the state-of-the-art CIMB-YOLO method. On the AI-TOD dataset, MSCM-YOLO improves mAP50 by 3.3 percentage points compared to the second-best model, HiDRA-DCDNet. This consistently superior performance across datasets with distinct characteristics demonstrates the robustness and generalization capability of MSCM-YOLO.

## 5. Visualization

**Figure 9:**
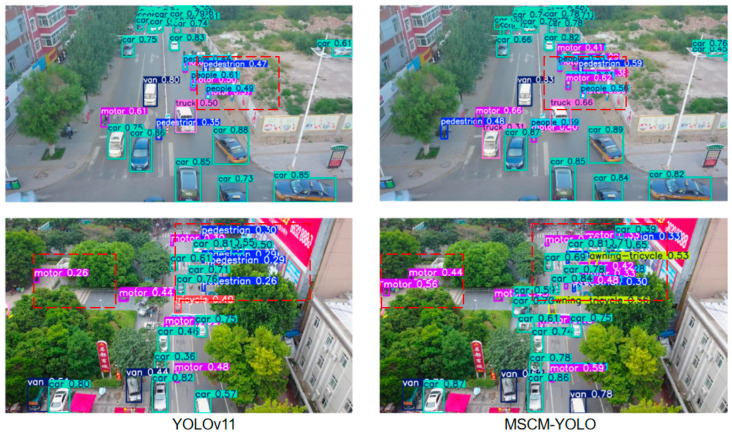
Detection Results of MSCM-YOLO and Baseline Models on the VisDrone2019 Dataset.

**Figure 10:**
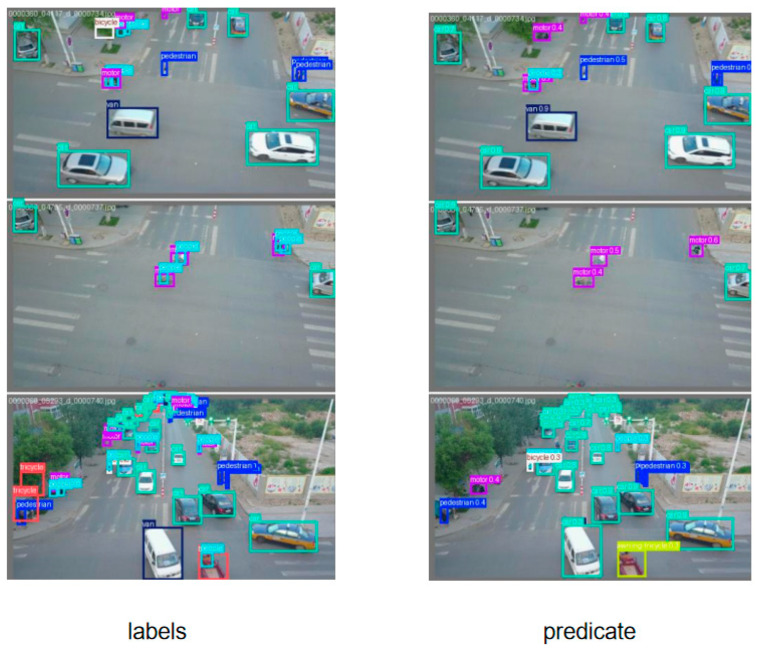
Detection Results of MSCM-YOLO on the VisDrone2019 Dataset.

To intuitively demonstrate the detection capability of the proposed MSCM-YOLO model, we conduct visualization experiments on the VisDrone2019 dataset and compare MSCM-YOLO with representative baselines under diverse challenging scenarios. The results are shown in Figure 9. In the detection result comparison, we focus on localization quality, missed detections of tiny objects, and false positives in cluttered backgrounds, particularly in dense scenes with severe occlusion and low-contrast targets. Complementarily, confusion-matrix analysis provides a class-wise view of misclassification patterns, especially among visually similar categories (Figure 10). By combining these two forms of analysis, the visualization experiments offer both intuitive evidence of improved robustness and clearer insights into how MSCM-YOLO mitigates category confusion under challenging VisDrone2019 conditions, thereby improving reliability for practical UAV-based surveillance applications.

### 5.1. Comparison of Detection Results

In the detection result comparison, MSCM-YOLO demonstrates improved localization and fewer missed detections for small targets, particularly in dense and heavily occluded scenarios. The comparison of detection results is shown in Figure 9.

As observed in Figure 9, the regions highlighted by the red dashed boxes show clear differences between MSCM-YOLO and YOLOv11. In these challenging areas, MSCM-YOLO detects small objects more reliably and produces tighter bounding boxes with more stable confidence scores, while reducing missed detections that frequently occur in the baseline. These results indicate that MSCM-YOLO better preserves fine-grained cues and suppresses background interference, enabling more accurate separation of true targets from visually similar patterns.

Moreover, MSCM-YOLO shows distinct advantages in dense regions where multiple targets are closely packed, as it maintains clearer instance separation and reduces duplicate boxes and false positives caused by crowding. It also performs better under severe occlusion (e.g., partially blocked pedestrians or vehicles), where the baseline often fails to respond. By leveraging enhanced contextual cues, MSCM-YOLO localizes partially visible instances more reliably, leading to improved recall and overall robustness in complex UAV scenes.

As shown in the detection results in Figure 10. Despite improved robustness, occasional false positives persist in high-texture backgrounds, and some extremely small, low-contrast targets remain undetected, highlighting directions for future refinement.

### 5.2. Confusion Matrix Analysis

**Figure 11:**
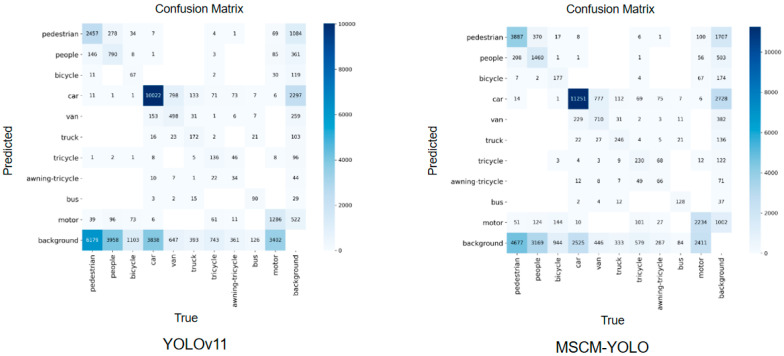
Confusion Matrix Comparison between MSCM-YOLO and YOLOv11.

As shown in Figure 11, the confusion matrix summarizes the correspondence between predicted labels and ground-truth categories for all object classes. The diagonal elements indicate correctly classified instances (true positives), reflecting the model’s recognition accuracy for each category, while the off-diagonal elements reveal misclassifications, highlighting which classes are most frequently confused. By examining both the magnitude and distribution of these entries, we can identify dominant error modes and assess whether errors are concentrated in specific categories or broadly distributed.

In the case of YOLOv11, there are a significant number of false positives, particularly related to the “background” class, indicating frequent target–background confusion in cluttered UAV scenes. For instance, there are 6070 false positives for pedestrians, suggesting the model often misidentifies background regions as pedestrians. Additionally, categories such as “van” and “truck” show substantial misclassification errors, reflecting difficulty in separating visually similar vehicle types.

MSCM-YOLO demonstrates a much more accurate classification across classes, with fewer false positives and a higher number of true positives overall. For example, the detection of “car” sees an increase in true positives (11,251), while false positives are reduced—especially for “pedestrian,” with only 4677 false positives—indicating improved suppression of spurious detections. This reflects better handling of target–background confusion and more reliable small-target localization in dense or occluded regions.

**Figure 12:**
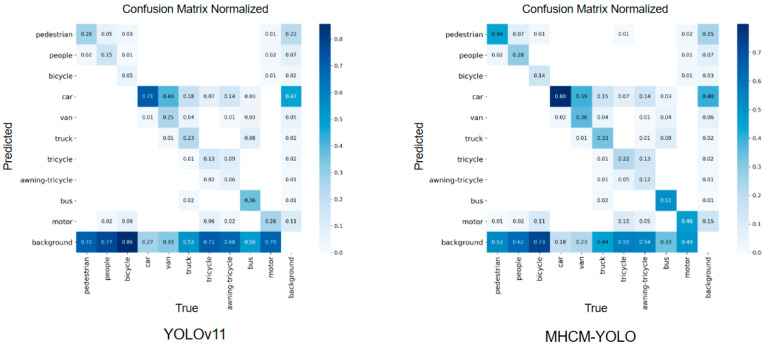
Confusion Matrix Normalized Comparison between MSCM-YOLO and YOLOv11.

As shown in Figure 12, the normalized confusion matrix displays the proportion of predictions for each ground-truth class, providing a scale-independent view of classification performance across categories. The values along the diagonal represent the percentage of correctly classified instances for each category, thereby directly reflecting per-class recognition accuracy. In contrast, the off-diagonal entries indicate the relative frequency of confusion between different classes, making it easier to pinpoint which categories are prone to being misidentified as others. In the visualization, a darker shade corresponds to a higher proportion (stronger diagonal performance), while darker off-diagonal cells suggest more prominent confusion patterns and systematic errors.

YOLOv11 exhibits inferior performance for smaller objects such as pedestrians, people, bicycles, and tricycles, as reflected by their notably lower diagonal values in the confusion matrix (e.g., pedestrian: 0.28, people: 0.15, bicycle: 0.05, tricycle: 0.13), which suggests substantial inter-class confusion. In contrast, MSCM-YOLO demonstrates a more concentrated diagonal distribution and superior inter-class discriminability. The diagonal entries for these small-object categories are markedly improved: pedestrian increases from 0.28 to 0.44, people from 0.15 to 0.28, bicycle from 0.05 to 0.14, and tricycle from 0.13 to 0.22. Notably, MSCM-YOLO also maintains high accuracy for larger objects (e.g., the “car” class achieves 0.80). These consistent improvements indicate that MSCM-YOLO effectively mitigates misclassification for small and occluded targets, leading to higher per-class accuracy and more reliable detection performance.

In contrast, MSCM-YOLO demonstrates substantially enhanced performance for small-object categories. This is directly evidenced by the marked increase in their diagonal confusion matrix values (e.g., pedestrian: 0.44, bicycle: 0.14). Notably, this improvement is achieved without compromising the detection of larger objects, as seen in the sustained high accuracy for the ‘car’ class (0.80). The overall reduction in inter-class confusion underscores the efficacy of MSCM-YOLO’s design in extracting discriminative features for challenging small and often occluded targets.

## 6. Conclusions

Vision-based UAV systems have become instrumental in aerial monitoring; however, reliable small object detection in UAV imagery remains a significant technical challenge due to several compounding factors: substantial scale variations induced by altitude and viewpoint changes; low-resolution and low-contrast targets with weak visual signatures—often comprising only a few pixels and degraded by motion blur and sensor noise; dense and frequently occluded object distributions; and cluttered backgrounds containing challenging visual artifacts such as shadows, textures, and reflections. A fundamental limitation of this work is that it does not explicitly address occlusion, which is prevalent in oblique aerial views. A UAV could actively avoid occlusion through flight-path planning—a promising future direction that is beyond the scope of single-frame detection.

Recent advances have aimed to improve small object detection in UAV imagery through multi-scale feature pyramid networks, attention-based feature refinement, lightweight backbone architectures, and contextual fusion mechanisms. While these approaches yield measurable gains, several limitations persist: inconsistent detection of extremely small, densely clustered objects under occlusion; sensitivity to background clutter and abrupt scale changes; and computational overhead from complex attention or aggregation modules that can hinder deployment on resource-constrained UAV platforms. Consequently, achieving robust detection in complex real-world aerial scenarios remains an open problem.

To address these challenges, this paper introduces MSCM-YOLO, a lightweight yet effective framework for UAV small object detection, built upon YOLOv11 with a multi-stage collaborative enhancement design. The proposed method integrates four key innovations: (i) a dedicated high-resolution P2 detection head to preserve fine-grained features for extremely small and crowded objects; (ii) a lightweight backbone enhanced with Mobile Bottleneck Convolution (MBConv) to strengthen feature extraction for low-visibility targets; (iii) a Scale-Adaptive Attention Fusion with Channel-Adaptive Projection (SAF+CAP) module for adaptive multi-level spatial–semantic feature integration under large-scale variations; and (iv) a Multi-Domain Feature Attention Fusion (MDFAF) module that jointly leverages spatial, frequency, and positional information to improve target–background discrimination in complex UAV scenes.

Comprehensive experiments on the VisDrone2019 dataset demonstrate that MSCM-YOLO achieves mAP50 and mAP50:95 scores of 44.41% and 27.13%, respectively, surpassing the YOLOv11 baseline by 10.77 and 7.22 percentage points. The framework achieves this significant performance advancement while maintaining computational characteristics appropriate for UAV deployment scenarios. Further evaluations on the UAVDT, DIOR, and AI-TOD datasets confirm consistent improvements in mAP50, underscoring the method’s robustness and generalization capability across diverse aerial monitoring scenarios.

In summary, MSCM-YOLO, through a carefully designed balancing scheme, effectively addresses the key bottleneck of small target detection on UAVs, enhancing high-resolution feature preservation, multi-scale feature representation, and the ability to distinguish targets from backgrounds. Its continuously improving performance across multiple benchmark tests, along with its practical design considerations, makes this framework a feasible and effective solution for real-world UAV aerial surveillance tasks. Future work will explore the following aspects: 1. full-resolution processing to better preserve the ultra-fine details of extremely small targets; 2. active vision strategies (e.g., viewpoint/flight path planning) to mitigate occlusion issues from the perspective of tilted UAVs; 3. furthermore, we will further develop a more adaptive multi-scale fusion mechanism to improve the framework’s robustness in abrupt scale changes and densely cluttered aerial scenarios, while maintaining its efficiency in airborne deployment.

## Figures and Tables

**Table 1:** Configuration of training and testing experiment environments.

Environment	Parameters
OS	Ubuntu 20.04
CPU	Intel(R) Xeon(R) Silver 4210R
GPU	NVIDIA GeForce RTX 3090
Language	Python 3.12
CUDA Version	CUDA 12.2
Framework	Torch 2.5.1

**Table 2:** Experimental parameters.

Imgsz	Epochs	Batch	Optimizer	Lr0	Momentum	Weight_Decay	Seed
640 × 640	300	8	SGD	0.04	0.937	5 × 10^−4^	42

**Table 3:** Results of ablation study on the VisDrone2019 dataset.

Variant	P2	MBConv	SAF+CAP	MDFAF	P(%)	R(%)	mAP50(%)	mAP50:95(%)	Param(M)	FPS
A					44.03	33.32	33.64	19.91	2.59	598.73
B	√				48.40	35.90	37.47	22.72	2.67	550.01
C	√	√			47.55	36.16	37.30	22.65	2.67	524.20
D	√		√		48.56	38.31	39.44	23.99	2.97	399.79
E	√			√	52.76	40.72	42.83	26.53	3.69	331.03
F	√	√	√		50.77	37.66	39.48	23.91	2.97	295.06
G	√	√		√	52.54	41.34	43.22	26.54	3.69	258.54
H	√		√	√	52.27	42.25	44.18	27.32	3.91	223.66
I	√	√	√	√	53.42	43.37	44.41	27.13	3.97	167.81

**Table 4:** Comparison across different categories in the VisDrone2019 dataset.

Model	Param	mAP50(%)										
		ALL	Ped	Peo	Bic	Car	Van	Truck	Tri	Awn	Bus	Motor
ATSS	31.0	36.9	42.7	22.3	18.8	76.6	41.4	36.9	28.4	8.5	52.1	41.4
TOOD	31.8	41.0	41.5	31.9	19.2	81.4	46.5	39.6	31.8	14.1	53.5	50.5
YOLOv8	3.0	33.8	35.7	28.6	8.25	76.2	39.1	29.8	21.6	12.2	48.9	37.9
VFNet	33.5	41.3	41.8	25.4	20.0	80.4	47.4	41.7	35.1	15.5	57.0	48.8
YOLOv11	2.6	33.6	35.9	27.3	8.37	76.1	39.9	30.5	21.0	11.9	47.2	37.4
Gold-YOLO	-	31.3	32.3	26.3	6.9	74.3	36.9	26.6	20.1	11.5	44.3	33.7
YOLOv12	2.5	31.9	34.8	26.0	7.96	74.8	37.3	26.1	18.9	11.6	46.5	34.9
HRMamba-YOLO	33.5	38.9	37.5	26.8	21.6	68.2	46.8	41.6	30.9	19.9	58.9	36.8
RT-DETR-R18	19.9	42.5	44.9	39.2	18.8	81.7	48.3	36.2	32.0	15.9	54.9	53.5
DASSF-YOLO	-	39.6	44.6	36.8	14.4	81.0	44.4	31.2	24.7	16.1	52.5	45.9
RMH-YOLOv8n	1.3	42.4	-	-	-	-	-	-	-	-	-	-
SRM-YOLO	3.2	39.4	52.7	42.1	12.7	80.5	43.1	34.1	25.9	14.8	53.6	45.2
MSCM-YOLO	3.9	44.4	50.7	41.2	16.6	83.6	48.8	39.9	32.1	17.7	61.8	51.7

**Table 5:** Overall performance comparison with recent state-of-the-art models on VisDrone2019.

Model	Param(M)	P(%)	R(%)	mAP50(%)	mAP50:95(%)	FLOPs/G
HRMamba-YOLO	33.5	-	-	38.9	-	96.4
DASSF-YOLO	-	49.9	39.0	39.6	23.5	-
RMH-YOLOv8n	1.3	53.0	40.4	42.4	25.7	16.7
SRM-YOLO	3.2	49.4	38.1	39.4	-	15.9
MSCM-YOLO	3.9	53.4	43.3	44.4	27.1	18.7

**Table 6:** Comparative results on DIOR and AI-TOD datasets.

Dataset	Model	F1(%)	mAP50(%)	mAP50:95(%)
DIOR	ATSS	82.9	83.1	52.1
	YOLOv8	82.0	84.5	62.1
	YOLOv11	82.5	85.2	63.0
	YOLOv12	82.9	84.8	62.3
	DASSF-YOLO	81.2	81.4	59.2
	CIMB-YOLO	83.9	85.3	63.2
	MSCM-YOLO	84.7	88.2	66.2
AI-TOD	YOLOv8	46.7	41.3	18.0
	YOLOv11	46.6	41.0	17.6
	FFCA-YOLO	45.8	36.9	16.0
	HGNetv2	44.4	39.5	17.4
	LeYOLO	43.2	37.5	16.0
	HiDRA-DCDNet	50.3	45.0	19.3
	MSCM-YOLO	54.4	48.3	21.6

## Data Availability

The datasets analyzed in this study are publicly available. These data were derived from the following resources available in the public domain: (1) VisDrone2019: https://github.com/VisDrone/VisDrone-Dataset#download (accessed on 27 February 2026); (2) UAVDT: https://sites.google.com/view/daweidu/projects/uavdt (accessed on 27 February 2026); (3) DIOR: https://drive.google.com/drive/folders/1UdlgHk49iu6WpcJ5467iT-UqNPpx__CC (accessed on 27 February 2026); (4) AI-TOD: https://github.com/jwwangchn/AI-TOD (accessed on 27 February 2026); No new dataset was created in this study. Additional materials (e.g., trained model weights and detailed training logs) can be made available by the authors upon reasonable request.
